# Prescriptions of Essentially Placebo Treatments Among General Practitioners in 21 Countries

**DOI:** 10.1001/jamanetworkopen.2025.32672

**Published:** 2025-09-18

**Authors:** Fabian Wolters, Kaya Peerdeman, Jacobijn Gussekloo, Limor Adler, Radost Asenova, Petra Bomberová Kánská, Claire Collins, Gheorghe Gindrovel Dumitra, Jeremy Howick, Sanda Kreitmayer Peštić, Donata Kurpas, Vanja Lazic, Heidrun Lingner, Christian D. Mallen, Aristea Missiou, Lieve Peremans, Ferdinando Petrazzuoli, Liina Pilv-Toom, Rosalinde K.E. Poortvliet, Hans O. Thulesius, Péter Torzsa, Rosy Tsopra, Victoria Tkachenko, Rita Viegas, Andrea W. M. Evers, Sven Streit

**Affiliations:** 1 Unit Health, Medical and Neuropsychology, Leiden University, Leiden, the Netherlands; 2 Leiden Institute for Brain and Cognition, Leiden University, Leiden, the Netherlands; 3 LUMC Center for Medicine for Older People, Department of Public Health and Primary Care, Department of Internal Medicine, Leiden University Medical Center, Leiden, the Netherlands; 4 Faculty of Medicine, Tel Aviv University, Tel Aviv, Israel; 5 Department of Urology and General Practice, Faculty of Medicine, Medical University of Plovdiv, Plovdiv, Bulgaria; 6 Department of Social Medicine, Charles University–Faculty of Medicine in Hradec Králové, Hradec Králové, Czech Republic; 7 Irish College of General Practitioners, Dublin, Ireland; 8 Department of Public Health and Primary Care, Ghent University, Ghent, Belgium; 9 University of Medicine and Pharmacy of Craiova, Craiova, Romania; 10 Stoneygate Centre for Empathic Healthcare, Leicester Medical School, University of Leicester, Leicester, United Kingdom; 11 JZNU Dom zdravlja “Dr. Mustafa Šehović” Tuzla, Tuzla, Bosnia and Herzegovina; 12 Division of Research Methodology, Department of Nursing, Faculty of Nursing and Midwifery, Wroclaw Medical University, Wrocław, Poland; 13 Health Center Zagreb–Centar, Zagreb, Croatia; 14 Center for Public Health and Healthcare, Department of Medical Psychology, German Center for Lung Research (DZL)/BREATH Hannover, Hannover Medical School, Hannover, Germany; 15 Keele University School of Medicine, Keele, Staffordshire, United Kingdom; 16 Research Unit for General Medicine and Primary Health Care, Faculty of Medicine, School of Health Sciences, University of Ioannina, Ioannina, Greece; 17 Department of Family Medicine and Population Health, University of Antwerp, Gouverneur Kinsbergen Centrum, Antwerp, Belgium; 18 Center for Primary Health Care Research, Department of Clinical Sciences, Lund University, Malmö, Sweden; 19 Institute of Family Medicine and Public Health, University Tartu, Tartu, Estonia; 20 Department of Medicine and Optometry, Faculty of Health and Life Sciences, Linnaeus University, Kalmar, Växjö, Sweden; 21 Department of Family Medicine, Faculty of Medicine, Semmelweis University, Budapest, Hungary; 22 Université Paris Cité, Sorbonne Université, Inserm, Centre de Recherche des Cordeliers, Paris, France; 23 Department of Medical Informatics, AP-HP, Hôpital Européen Georges-Pompidou, Paris, France; 24 Department of Internal Medicine of Postgraduate Education and Training Centre of Family Medicine, Bogomolets National Medical University, Kyiv, Ukraine; 25 Department of Family Medicine, NOVA Medical School, Lisboa, Portugal; 26 Department of Psychiatry, Leiden University Medical Center, Leiden, the Netherlands; 27 Medical Delta, Erasmus University Rotterdam, Leiden University & Delft University of Technology, Delft, the Netherlands; 28 Institute of Primary Health Care (BIHAM), University of Bern, Bern, Switzerland

## Abstract

**Importance:**

General practitioners (GPs) sometimes initiate a treatment despite not expecting it to improve patients’ symptoms by any physiological mechanism. These essentially placebo treatments are ethically controversial, and their frequency is unclear. They involve risks for patients, but to estimate these, more data are needed.

**Objective:**

To develop a more precise overview of the rate at which GPs prescribe essentially placebo treatments.

**Design, Setting, and Participants:**

This cross-sectional survey study included currently practicing GPs from 20 European countries and Israel who responded to online questionnaires. The online questionnaires were taken between December 12, 2019, and August 4, 2021, and analyzed on April 28, 2022. Respondents were contacted by national representatives, either through personal networks (convenience sampling) or an existing database (volunteer sampling).

**Main Outcomes and Measures:**

The main outcome was the rate of essentially placebo prescriptions, given as the rate per week and the proportion of consultations. Secondary outcomes were the associations between this rate and GP background characteristics (gender, age, education about placebos, years of experience, patients seen, and working hours per week).

**Results:**

A total of 952 practicing GPs responded (453 of 745 [61%] female; mean [SD] age of 48.02 [11.95] years), and 669 answered all questions. Overall, 689 of 818 respondents (84%) indicated they had prescribed an essentially placebo treatment at least once. Overall, the median (IQR) rate of essentially placebo prescriptions was 0.5 (0.1 to 2.0) per week or 0.67% (0.06% to 2.50%) of consultations. The prescription rate was higher in men (β = 1.94 [95% CI, 0.58 to 3.29]; *P* = .005), those with more work experience (β = 0.12 [95% CI, 0.06 to 0.18]; *P* < .001), and those who work fewer hours per week (β = −0.08 [95% CI, −0.13 to −0.03]; *P* = .001).

**Conclusions and Relevance:**

In this survey study of GPs across 21 countries, essentially placebo prescriptions featured in a small minority of consultations, but they nevertheless occurred regularly for most GPs. Rates varied only slightly by GP background characteristics. This suggests that essentially placebo prescriptions were common at a population level, which poses risks for the patient-GP relationship and creates medical risks for patients. Future research should further investigate the decision-making process behind these prescriptions and their effects on patients.

## Key Points

**Question:**

How often do general practitioners (GPs) prescribe a treatment that is essentially a placebo?

**Findings:**

In this survey study with 952 participants across 21 countries, the median prescription rate for treatments that GPs do not expect to improve symptoms via physiological mechanisms was once per 2 weeks or in 0.7% of all consultations. Although few differences by GP characteristics were found, male GPs and those with more work experience were significantly more likely to prescribe these treatments.

**Meaning:**

While the rate of prescribing essentially placebo treatments in this study was relatively low per GP, it did amount to a relevant number of prescriptions at a population level, raising concerns about GP-patient relationships and potential adverse effects.

## Teaser

This survey study evaluates how often general practitioners prescribe essentially placebo treatments, that is, treatments for which any positive effect on symptoms cannot be attributed to the treatment’s pharmacological or biological components.

## Introduction

Physicians sometimes prescribe a treatment even when they do not expect it will help the patient in any pharmacological or biological way—essentially, prescribing a placebo.^1,2,3,4,5,6,7,8,9^ Current estimates of the proportion of GPs who have given these prescriptions at least once vary widely, from 29% to 97%.^10^ Existing studies were performed in a variety of different countries and included physicians with a variety of background characteristics. The definition of a placebo prescription also varied. Some studies made a distinction between pure placebos—those with no active ingredients, such as a sugar pill—and impure placebos—which do have an active ingredient but will not help resolve the current concerns of the patient,^5,9^ such as an antibiotic for a viral infection. In these studies, different outcomes were found for pure and impure placebos, but the distinction has also been subject to critique.^11,12^ As such, we will use the term *essentially placebo*, which encompasses both pure and impure placebos.

While physicians may have good reasons for prescribing a treatment that is essentially a placebo, such as avoiding conflict or reassuring an anxious patient,^1,13,14^ they are nonetheless legally and ethically dubious.^15^ Most physicians do not inform the patient they are essentially prescribing a placebo^2,3,4,5,8^ and instead seem to leave this vague intentionally.^13^ Because many of these placebos still have active ingredients, they can also expose the patient to adverse effects.^16^ Accordingly, many medical guidelines categorically advise against placebo prescriptions in clinical practice.^16,17^

General practitioners (GPs) seem to prescribe essentially placebo treatments to maintain a good doctor-patient relationship. However, this relationship can also suffer harm if the use of an essentially placebo prescription is discovered. Because the current estimates of the rate of these prescriptions are uncertain, prescribing essentially placebo treatments is potentially a large risk. The risks are especially acute for patients who have a higher chance of receiving these treatments, such as patients with unexplained symptoms. A more precise estimate of the prescription rate would allow us to determine the size of the risk and the need for interventions, such as education about alternatives. To achieve this, we surveyed GPs from 21 mostly European countries about the rate at which they prescribe essentially placebo prescriptions and their background characteristics, using the same phrasing and definitions.

## Methods

### Design

In this survey study, respondents completed a single online questionnaire that took around 10 minutes to complete. This study was reviewed by the Medical Ethical Committee Leiden-Den Haag-Delft and the Leiden University data officer for the original version, and by the Keele University Faculty of Medicine and Health Sciences Research Ethics Committee Review for the UK version. All respondents provided informed consent. The survey was performed and is reported here in accordance with American Association for Public Opinion Research (AAPOR) reporting guideline.^21^

### Setting

Data were collected through the European General Practice Research Network (EGPRN).^18,19,20^ For each participating country, a national representative recruited participants, arranged a translation (if necessary), and functioned as a pilot respondent.

Data collection occurred at different times in 21 countries, between December 12, 2019, and August 4, 2021. Questionnaires were open for approximately 1 month in each country. eTable 1 in Supplement 1 shows the time of data collection by country.

### Questionnaire

The questionnaire was designed by an international and interdisciplinary team consisting of various disciplines (medical psychology, general practice, biomedical research, philosophy, ethics). The initial version of the questionnaire was drafted by psychologists (F.W., K.P., and A.W.M.E.) experienced with this type of survey, then reviewed by the rest of the team, and then reviewed again by the national representatives of each participating country. In each case, questions were adjusted to ensure uniform understanding and face validity.

In addition to original questions, several example cases and questions about them were taken from existing questionnaires^8,9^ to allow for direct comparison. The full questionnaire appears in the eAppendix in Supplement 1. Participants could choose not to answer any questions except for the frequency of prescriptions and still proceed, and they could exit at any time. This gave the respondents the option of not answering questions when they were unsure and kept them engaged throughout.

To avoid bias, participants were introduced to the questionnaire with a few example cases that were presented without any indications of their acceptability and without mentioning the term *placebo*. After filling in this part of the survey, respondents were given a uniform definition of essentially placebo treatments:

We consider a treatment as **essentially placebo** when, in your estimation, any positive treatment effect on the patient’s symptoms is **not** caused by the pharmacological or biological components of the treatment.

Respondents were then asked how often they prescribed this type of treatment on average, indicating a number and a timescale (per week, per month, per year, or per 10 years).

Background characteristics were then collected: gender, age, years of experience practicing as a GP, average number of patients seen per week, and working hours in clinical practice per week. Respondents were also asked to rate their perception of the quality of their education on using placebos in clinical practice on a visual analog scale, with the extremes indicating “completely insufficient” and “completely sufficient.” The questionnaire was completed online through Qualtrics XM (Qualtrics).

### Participants

The national representatives contacted as many colleagues as possible from the country they lived in, either through personal networks or an existing database. This meant that our sample was not probability-based and national samples are likely not representative, especially smaller samples based on personal networks, which are likely biased by location. The only criterion was that respondents were working as a GP at the time of responding, and they were reminded of this when starting the questionnaire. Participants were invited through email or in person. A reminder was sent 1 week after the original invite and again if response was lagging.

Before answering any questions, respondents received information about the study, the kind of data being collected and assurance of anonymity on the first page of the questionnaire. They could then provide informed consent.

### Statistical Analysis

The main outcome was the prescription rate of essentially placebo prescriptions, standardized to estimated weekly prescriptions. This number was then divided by the number of patients seen per week to calculate the percentage of consultations in which an essentially placebo prescription was given. Frequency was also calculated as a dichotomous variable (ever prescribed or never) for comparison with existing studies. To analyze whether individual characteristics of the GPs were associated with the frequency of placebo prescriptions, the frequency per patient was also entered as an outcome in a multivariable linear regression analysis. Gender, age, perceived quality of education about placebo effects, years of experience, average number of patients seen per week, and average working hours per week were entered as factors. The method of respondent acquisition (personal network or existing database) was also added as a factor to examine a possible confounding effect. Only the 669 respondents with a valid score on each variable used in the regression were included in this analysis. All analyses were performed in SPSS statistical software version 29 (IBM Corp). An α level of .05 (2-sided where applicable) was used for all tests, but a Bonferroni correction was applied to the tests of the final 6 individual factors of the regression (α = .05/6 = .0083) as a conservative approach considering multiple tests and a high number of participants.^22^ Because the number of countries was large and the samples from some were relatively small, a statistical comparison between countries would not be meaningful. Therefore, neither the rate of essentially placebo prescriptions nor the association of individual characteristics with this rate were compared in this way. Initial analysis was performed April 28, 2022.

## Results

### Participants

Overall, 952 respondents participated (453 of 745 [61%] female; mean [SD] age of 48. 02 [11.95]); 669 answered every question involved in the analyses reported here. The largest possible number of responses was used for every analysis, which means the effective number varies. The characteristics of the subsamples used for each analysis are shown in eTable 2 in Supplement 1. Differences between these samples were small. Sample characteristics based on the number of valid responses per variable appear in Table 1.

**Table 1.** Sample Characteristics of Participating GPs From 21 Countries

Variable	Respondents, No.	Mean (SD)
Gender, No. (%)		
Female	745	453 (61)
Male	745	292 (39)
Age, y	742	48.02 (11.95)
Years of practicing as a GP	741	16.93 (11.03)
Patients/wk, No.	738	120.54 (98.38)
Working h/wk in clinical practice	734	32.22 (14.72)
Reported feeling educated about essentially placebo treatments^a^	690	42.47 (27.10)
Recruitment strategy, No. (%)		
Existing database	952	537 (56)
Personal network	952	415 (44)

Abbreviation: GP, general practitioner.

^a^ The range for this scale was 1 to 100, with 1 indicating feeling the least educated and 100 indicating feeling the most educated.

Participants were recruited from 21 countries: Belgium (Flanders only), Bosnia and Herzegovina, Bulgaria, Croatia, the Czech Republic, Estonia, France, Germany, Greece, Hungary, Ireland, Israel, Italy, the Netherlands, Poland, Portugal, Romania, Sweden, Switzerland, the UK, and Ukraine. The number of participants, response rates, and prescription rates per country can be found in eTable 1 in Supplement 1.

### Types of Essentially Placebo Prescriptions

Answers to question 10 of the questionnaire (“Which treatments have you yourself given that would fall under the above definition of essentially placebo?”) were inspected to check whether respondents gave answers that matched our definition of essentially placebo treatments. While a full analysis of the varied answers is beyond the scope of this article, the most common was some type of vitamin, followed by other supplements and alternative medicine, such as homeopathy. Medications with (more) adverse effects, such as antibiotics or benzodiazepines, were only mentioned occasionally. Pure placebos, such as empty patches or saline solutions were rare, as were purely psychological approaches, such as reassurance. Overall, these results align with our definition and mirror outcomes from existing work,^4,8^ although in some earlier studies antibiotics appeared more often than in ours.^3,9^

### Rate of Essentially Placebo Prescriptions

A total of 818 respondents indicated how often they gave an essentially placebo prescription; 689 (84%) indicated that they had prescribed an essentially placebo treatment at least once in their professional career, varying from 56% in the UK to 100% in Ireland (eTable 1 in Supplement 1). A comparison between the group that had never prescribed an essentially placebo treatment and those who did so at least once is included in Table 2. Only the recruitment strategy and the prescription rate differed significantly between these groups.

**Table 2.** Sample Characteristics of Participating GPs by Prescription History

Variable	Never prescribed (n = 129)		Ever prescribed (n = 689)		*P* value
	Respondents, No.	Mean (SD)	Respondents, No.	Mean (SD)	
Gender, No. (%)					
Female	120	78 (65)	625	375 (60)	.30^a^
Male	120	42 (35)	625	250 (40)	
Age, y	120	47.34 (11.84)	622	48.15 (11.97)	.50
Years of practicing as a GP	118	15.5 (9.87)	623	17.2 (11.22)	.13
Patients/wk, No.	119	109.45 (91.54)	619	122.68 (99.57)	.18
Working h/wk in clinical practice	118	30.11 (15.72)	616	32.62 (14.50)	.09
Feeling educated about essentially placebo treatments^b^	111	41.96 (31.13)	579	42.57 (26.29)	.85
Recruited via existing database, No. (%)	129	93 (72)	689	367 (53)	<.001^a^^,^^c^
Prescription rate as % of consultations, median (IQR)	129	0	619	1.00 (0.31-3.21)	<.001^c^

Abbreviation: GP, general practitioner.

^a^ Tested with χ^2^. All other comparisons used independent *t* tests.

^b^ The range for this scale was 1 to 100, with 1 indicating feeling the least educated and 100 indicating feeling the most educated.

^c^ Significant result.

In the full sample of 818 GPs, the median rate of placebo prescriptions was once per 2 weeks (median [IQR], 0.5 [0.1-2.0] per week). Among the 689 respondents who prescribed a placebo at least once, the median (IQR) rate was 1 (0.3-3.0) per week.

The rate of essentially placebo prescriptions per week was divided by the number of consultations per week to correct for differences in the number of patients seen by each respondent. This resulted in the proportion of essentially placebo prescriptions per consultation. The median (IQR) rate of prescriptions per consultation was 0.67% (0.06%-2.50%) overall (ie, once in every 150 consultations) and 1.00% (0.31%-3.21%) among those who prescribed a placebo at least once. The Figure shows the full distribution, showing a strong right skew. Because not every person who indicated the rate of prescriptions also indicated the number of consultations per week, the effective sample size is slightly smaller for these rates (748 and 619, respectively).

**Figure.**
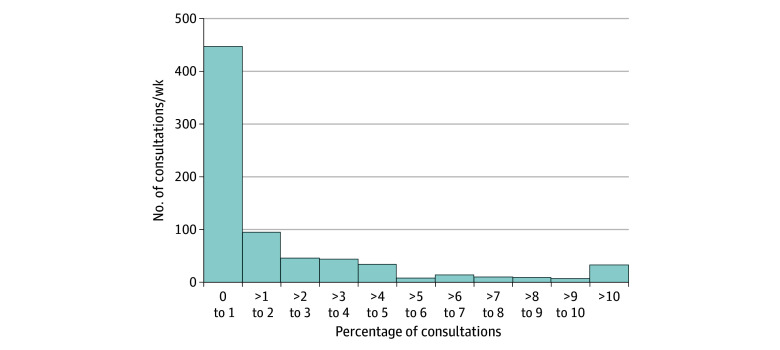
Histogram of the Percentage of Consultations in Which an Essentially Placebo Treatment Was Prescribed

The rate varied per country, from 0.1% in the UK to 2.5% in France, although these figures should be interpreted with caution because of the small sample size of some countries. An overview is available in the eFigure in Supplement 1.

### Prescribing Rate and Background Variables

To further investigate factors associated with prescription behavior, a linear regression was performed with background variables as factors and the per consultation rate of essentially placebo prescriptions as the outcome. Only the 669 participants responding to all relevant questions were included in this analysis. The list of factors and Pearson correlations between them are displayed in Table 3. Because of the high correlation between years practicing and age, collinearity was a likely risk. Years practicing was seen as the more relevant variable and kept for the final analysis, while age was removed.

**Table 3.** Correlations Between Regression Variables

Variables	Prescription rate	Gender	Age	Years practicing	Patients/wk	Working h/wk	Placebo education
Gender	0.12^a^	NA	NA	NA	NA	NA	NA
Age	0.14^a^	0.20	NA	NA	NA	NA	NA
Years practicing	0.15^a^	0.16^a^	0.87^a^^,^^b^	NA	NA	NA	NA
Patients/wk	−0.13^a^	−0.02	−0.07	−0.01	NA	NA	NA
Working h/wk	−0.13^a^	0.16^a^	0.03	0.01	0.31^a^^,^^b^	NA	NA
Placebo education	−0.05	−0.06	0.04	0.06	0.05	−0.01	NA
Recruitment strategy	0.06	0.10^a^	0.27^a^	0.19^a^	−0.16^a^	0.08^a^	0.09^a^

Abbreviation: NA, not applicable.

^a^ Significant correlation (bivariate).

^b^ Correlation of medium or large size (>0.30).

The whole model explained the rate of essentially placebo prescriptions (*R^2^* = 0.07; *P* < .001). The prescription rate was higher among male GPs (β = 1.94 [95% CI, 0.58 to 3.29]; *P* = .005), GPs with more years of experience practicing (β = 0.12 [95% CI, 0.06 to 0.18]; *P* < .001), and those who worked fewer hours per week (β = −0.08 [95% CI, −0.13 to −0.03]; *P* = .001) (Table 4). The contribution of each significant variable was small (all semi-partial correlations ≤.14).

**Table 4.** Results of Regression Analysis of Association of All Given Variables With the Rate of Essentially Placebo Prescriptions Per Consultation

Variable	β (95% CI)	*P* value
Gender (0 = female; 1 = male)	1.94 (0.58 to 3.29)	.005^a^
Years practicing	0.12 (0.06 to 0.18)	<.001^a^
No. of patients/wk	−0.01 (−0.014 to −0.00)	.06
Working h/wk in clinical practice	−0.08 (−0.13 to −0.03)	.001^a^
Feeling educated about essentially placebo treatments	−0.02 (−0.42 to 0.01)	.15
Recruitment strategy (0 = personal network; 1 = existing database)	0.65 (−0.72 to 2.01)	.35

^a^ Significant result (Bonferroni corrected α = .05/6 = .008).

An alternative regression including age and excluding years practicing yielded similar results, with age being a significant factor (β = 0.09; 95% CI, 0.04-0.15; *P* = .002). Statistical significance for other variables did not change. Similarly, when the regression was performed only on those respondents who prescribed an essentially placebo treatment at least once, results were highly similar, and no changes in statistical significance were observed.

## Discussion

In this study, we investigated the rate at which GPs reported they administered essentially placebo treatments. Examining the responses of 818 GPs across 21 countries, we found that 84% of respondents prescribed an essentially placebo treatment at least once in their career. On average, such prescriptions occurred once every 2 weeks or once in every 150 consultations. The rate of prescriptions also varied by country, ranging from 0.1% of consultations in the UK to 2.5% of consultations in France. Essentially placebo treatments were more often given by men, those who had more years of experience, or worked fewer hours per week, although all such differences were small.

Compared with existing studies, our results offer a more specific estimate of the placebo prescription rate (once every 2 weeks on average). Most studies have not investigated the exact rate of prescriptions beyond the at least once question.^6^ Those studies that have looked further used varying and generic indicators (eg, more than 10 times in the past year,^1^ quite often,^7^ once per week or month or less^4^). The most exact estimate^5^ indicated 2% of Swiss GP and pediatrician respondents prescribed placebos weekly and 5% daily. However, this concerned only the prescription of pure placebos, which in all studies appears to be less common than so-called impure placebos. Of course, our estimate is also potentially biased, since we relied on voluntary reporting of a non–evidence-based practice. Memory and social desirability both likely influence the results. However, there is no clear solution for this: the same bias against non–evidence-based practices would likely apply to prospective registration, and data extraction from electronic medical records is near impossible in this case because of the wide variety of products that can be prescribed as essentially placebo treatments. Our study therefore offers an important next step in quantifying the rate of the phenomenon of placebo prescriptions.

The diverse sample of GPs from different countries makes it likely that our results are more generalizable between countries than those of earlier studies. Indeed, the rate of respondents indicating they prescribed a treatment that was essentially placebo at least once (84%) in our study is comparable with that found in existing similar studies.^5,8,9^ The differences in rates between countries found in this study are also smaller than those found between different studies performed independently in different countries,^6^ showing the importance of keeping the phrasing of questions and definitions consistent.

One aspect in which our results differed from existing studies is the association between individual characteristics and prescribing rates. Previous work found no^2,3,4^ or little^9^ association with gender and age,^2,3,4^ while we found associations with gender and age as well as working hours per week and number of years of working experience. However, it should also be noted that the observed associations are relatively small and may thus not be observed in smaller studies^2,3^ or may even be chance findings. As such, more research is needed to offer an evidence-based profile of a typical prescriber of essentially placebo treatments.

The results of this study show that essentially placebo prescriptions are at the same time both frequent and infrequent: frequent because most GPs do prescribe them some of the time, and infrequent because they occur for only a small minority of consultations. The fact that each GP only prescribed an essentially placebo treatment infrequently may lead them to see such prescriptions as relatively harmless. However, the fact that this seems to apply across GPs—regardless of the characteristics of the GP in question—means that it does end up happening in a large number of consultations at the population level. From a patient’s perspective, a 1 in 150 chance of receiving essentially placebo medication may be worrying. Patients who discover that their medication was not prescribed for the reasons they thought may lose trust in their GP. This problem is compounded by the data suggesting that placebo prescriptions are more often given to reduce conflict with difficult patients^1,2,5,8,14^ or to treat patients who present with psychogenic complaints,^5,8,9,13^ whose relationship with their GP might already be strained. In such cases, an essentially placebo prescription could carry the risk of further alienation. Because most essentially placebo treatments still have active ingredients,^5,8,9^ they also carry the risk of adverse effects. As our data show that these prescriptions nevertheless still happen, there must be other motivations that outweigh these risks. We recommend further research into the decision-making process behind essentially placebo treatments and the risks and harms they may entail.

### Limitations

Our study has several important limitations. The response rate clearly varied between countries, and the recruitment method (which covaried with country) differed between groups who had never prescribed an essentially placebo treatment and those who had. This could indicate bias: for example, the questionnaire could have been less interesting to GPs from countries where essentially placebo prescriptions are less controversial, which would lead to an underestimation of the prescription rate. Participants recruited through a personal network might be more similar to the representative who recruited them than the national population, leading to inflated homogeneity. However, it should be noted that studies suggest that lower response rates are common in GP surveys and do not necessarily induce selection bias.^18,23,24,25^ We also included a relatively low number of participants per country, which means our data did not allow statistical comparisons between countries and likely did not offer a representative sample for each country, especially when participants were recruited through a personal network. Apart from Israel, only European countries are included, and we cannot generalize to practices in other countries. We therefore welcome more cross-national research in this area, especially outside of Europe.

## Conclusions

In this survey study of GPs from 21 countries, we found that while essentially placebo prescriptions feature in a small minority of consultations, they nevertheless occur regularly for most GPs. Future research should further investigate the precise decision-making process behind and treatment outcomes of these prescriptions.

## Notes

Supplement 1. **eFigure. **Median Essentially Placebo Prescription Rate as a Percentage of Consultations per Country **eTable 1. **Responses and Prescription Rates per Country **eTable 2. **Sample Characteristics of Participating GPs **eAppendix.** Full Questionnaire

Supplement 2.
Data Sharing Statement
