# Supplementary material for: Prescriptions of Essentially Placebo Treatments Among General Practitioners in 21 Countries
Source: JAMA Netw Open. 2025 Sep 18;8(9):e2532672. doi: 10.1001/jamanetworkopen.2025.32672 (PMC12447254; doi:10.1001/jamanetworkopen.2025.32672)
Supplement: Supplement 1. — eFigure. Median Essentially Placebo Prescription Rate as a Percentage of Consultations per Country eTable 1. Responses and Prescription Rates per Country eTable 2. Sample Characteristics of Participating GPs eAppendix. Full Questionnaire [file jamanetwopen-e2532672-s001.pdf]

Supplemental Online Content

Wolters F, Peerdeman K, Gussekloo J, et al. Prescriptions of essentially placebo treatments among general practitioners in 21 countries. *JAMA Netw Open.* 2025;8(9):e2532672. doi:10.1001/jamanetworkopen.2025.32672

eFigure. Median Essentially Placebo Prescription Rate as a Percentage of Consultations per Country

eTable 1. Responses and Prescription Rates per Country

eTable 2. Sample Characteristics of Participating GPs

eAppendix. Full Questionnaire

This supplemental material has been provided by the authors to give readers additional information about their work.

© 2025 Wolters F et al. *JAMA Network Open*.

eFigure. Median Essentially Placebo Prescription Rate as a Percentage of Consultations per Country

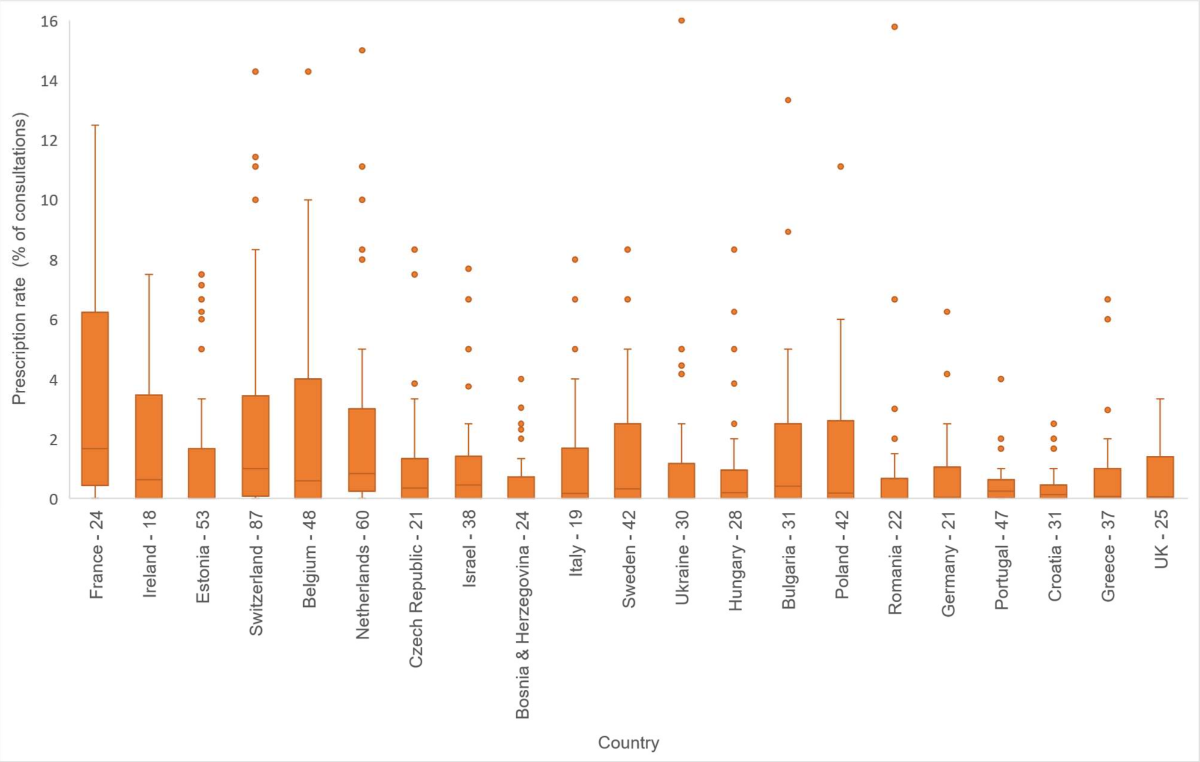

Note. Countries are listed in descending order of median prescription rate. The middle line indicates the median, ends of boxes indicate interquartile ranges, whiskers indicate outer quartiles ignoring outliers, dots indicate outliers. Some extreme outliers are not pictured to preserve legibility. Numbers by each country indicate the number of valid responses.

© 2025 Wolters F et al. *JAMA Network Open*.

eTable 1. Responses and Prescription Rates per Country

Country	Start date of survey administration	Number of responses	Number of GPs contacted	Response rate	Percent ever prescribed	Median prescription rate (% of consultations)
Belgium/Flanders	27/05/2021	60	Unknown		92.6%	1.1%
Bosnia and Herzegovina	30/06/2020	47	50	94%	79.3%	0.7%
Bulgaria	27/04/2021	35	45	77.8%	79.4%	0.5%
Croatia	23/06/2021	35	61	57.4%	84.9%	0.2%
Czech Republic	18/05/2021	28	95	29.5%	95.7%	0.8%
Estonia	18/06/2021	91	800	11.4%	84.4%	1.3%
France	15/07/2020	25	43	58.1%	91.7%	2.5%
Germany	13/10/2020	26	439	5.9%	91.3%	0.3%
Greece	18/01/2020	46	90	51.1%	73.2%	0.2%
Hungary	09/06/2021	41	Unknown		91.2%	0.6%
Ireland	04/03/2020	26	500	5.2%	100%	2.4%
Israel	01/26/2020	50	350	14.3%	83.7%	0.8%
Italy	19/01/2020	29	40	72.5%	85.7%	0.7%
Netherlands	14/07/2020	65	372	17.5%	87.1%	1%
Poland	12/12/2019	48	67	71.6%	75%	0.5%
Portugal	19/07/2020	54	88	61.4%	90.2%	0.3%
Romania	17/02/2020	31	50	62%	60.9%	0.4%
Sweden	14/06/2021	46	174	26.4%	79.6%	0.7%
Switzerland	04/09/2020	97	269	36.1%	94.6%	1.3%
UK	20/01/2020	26	42	61.9%	56%	0.1%
Ukraine	22/06/2021	46	52	88.5%	70.6%	0.7%
**Total**		**952**	**3627**	**Median 57.4%**	**Mean 83.2**	**Median 0.7**

© 2025 Wolters F et al. *JAMA Network Open*.

eTable 2. Sample Characteristics of Participating GPs

Variable	Largest n (N=952)		Indicated prescription frequency (n=818)		Regression analysis (n=668 for all variables)
	n	mean (SD), median, or %	n	mean (SD), median, or %	mean (SD), median, or %
Gender	745	61% female	745	61% female	59% female
Age (years)	742	48.02 (11.95)	742	48.02 (11.95)	48.16 (11.74)
Years of practicing as a GP	741	16.93 (11.03)	741	16.93 (11.03)	16.98 (10.82)
Average number of patients per week	738	120.54 (98.38)	738	120.54 (98.38)	119.8 (96.27)
Working hours per week in clinical practice	734	32.22 (14.72)	734	32.22 (14.72)	32.36 (14.78)
Feeling educated about essentially placebo treatments (1-100)	690	42.47 (27.1)	690	42.47 (27.1)	42.23 (27.02)
Recruitment strategy (existing database/personal network)	952	57% existing database	818	56% existing database	55% existing database
Prescription rate as percentage of consultations	748	median 0.67%	748	median 0.67%	median 0.67%

© 2025 Wolters F et al. *JAMA Network Open*.

eAppendix. Full Questionnaire

When working as a general practitioner (GP), you may be presented with difficult cases. Some patients may suffer from a condition for which no treatment is available. In other cases, the patient has certain complaints, but medical tests do not show signs of any medical condition. And some patients insist on a specific treatment, even if it is not indicated for their complaint. We know from previous studies that in cases like these, GPs sometimes give a treatment even when they do not think the treatment actually contains a pharmacological or biological component that would help with their patient's complaint. Of course, maybe the patient still feels better after receiving the treatment; an anxious patient might be reassured, or the treatment could trigger a placebo effect.

This study aims to investigate exactly how often these kinds of treatments are initiated, and for what reasons. Your answers can help us understand what the best course of action is in these complex cases, and how GPs can be best supported in making the right decisions.

The survey will take approximately 5-10 minutes to complete.

This international survey is the result of a collaboration between researchers at Leiden University and Leiden University Medical Center (the Netherlands), the University of Oxford (UK), and the University of Bern (Switzerland). If you have any questions about participation or the survey in general, please contact us.

Kind regards,

[name of national coordinator] [email address of national coordinator]

Dr. Sven Streit, MD

Prof. Andrea Evers and Prof. Jacobijn Gussekloo, the Netherlands

Prof. Jeremy Howick

Please note: this survey is intended for general practitioners. If you are not currently working as a general practitioner, please refrain from participating.

This survey is entirely anonymous. We will not collect personal information, and it will not be possible to connect you to your answers, either by the research team or by third parties. Your answers will only be used for scientific purposes. Your anonymous data will be saved for a period of at least 15 years.

Do you consent to participating in this survey and your data being used in the way described above?

□ Yes □ No

© 2025 Wolters F et al. *JAMA Network Open*.

Case studies

We will start the survey with 4 **case studies**. Each case is followed by a few questions. Imagine the cases as if they concerned your patients, and respond as you would in practice.

Case 1. A healthy forty-year-old self-employed male visits you in your practice, complaining of a cold and cough lasting for 5 days. He would like you to prescribe the medication he received the last time he had a cold, so he can go back to work again as soon as possible. The patient insists as this treatment had supposedly helped him very well during the last cold.

Would you prescribe the previously given medication, assuming records show that it was…

1. … a low (10 mg) dose of codeine, that is unlikely to greatly affect the patient's symptoms?

□ Yes □ No

2. … antibiotics, but the patient now most likely has a viral infection?

□ Yes □ No

3. … multivitamin pills?

□ Yes □ No

4. … sugar pills with no pharmacological effects?

□ Yes □ No

Case 2. A seventy-year-old female comes in to your practice. You know her from previous visits to be a demanding patient who is very worried about her health. The patient reports that she recently fell when she got up quickly from bed at night. Although there were no sequelae, she is afraid to fall again and has limited her activities She is not on any medication that would cause this, nor does your examination indicate other probable causes. The patient requests iron supplements, having heard somewhere that they can help with dizziness. She insists even when you explain that you have noticed no signs of iron deficiency.

5. How do you proceed with this patient?

□ I reassure her by telling her there is nothing wrong, without giving a prescription or reference.
□ I prescribe the iron supplements.
□ I refer her to a specialist to address her fears.

Case 3. Imagine you have been prescribing a certain decongestant nasal spray as a treatment for patients with sinusitis for several years. Prevailing medical opinion at the time that you started prescribing the spray was that it was an effective short-term treatment, and indeed the patients that you have given it to over the years all seem to have improved after using the spray as indicated. However, a new randomized clinical trial, published in the Lancet, shows that the treatment performs no better than placebo. By all indications, the study is a more stringent and careful test than previous studies of this treatment.

6. How do you respond to this study in terms of prescribing this decongestant nasal spray to patients with sinusitis?

□ I will continue to prescribe it as before.
□ I will continue to prescribe it, but only to patients similar to those it has worked for in the past.
□ I will continue to prescribe it, but only to the specific patients it has worked for in the past.
□ I will continue to prescribe it, but only if no other treatment is possible.
□ I will stop prescribing it.

Case 4. A patient in a hospital with severe postoperative pain receives an analgesic through an

© 2025 Wolters F et al. *JAMA Network Open*.

infusion pump. The pump releases the analgesic 3 times per day automatically, but the patient can also press a button to receive it on demand. However, the pump has built-in limits that prevent the release of the analgesic when the button is pressed too often or too close to another release. The patient is unaware of whether a release is triggered or not. He reports that his pain is reduced whenever he presses the button, even though the pump did not release an analgesic in many cases.

7. What conclusion(s) can you draw from the fact that pain is reduced even in those cases when no analgesic is released? Check all that apply.

◻ The pain had no organic cause, but was psychogenic.
◻ The pain was not particularly strong.
◻ The patient is very suggestible.
◻ The intensity of pain decreased naturally.
◻ The patient gave the answer they think you want to hear.
◻ The patient's pain was reduced by a placebo effect.
◻ The pain reduction was conditioned (learned; a Pavlovian response occurred).
◻ The expectation of pain reduction has led to the decrease in pain.
◻ The positive attention by the nursing staff led to a decrease in pain.
◻ Other (please specify):___________________________________

© 2025 Wolters F et al. *JAMA Network Open*.

General questions

The introduction of this survey and the cases you have just read describe a certain type of treatment. For the purpose of this survey, we will call this type of treatment **‘essentially placebo’**, and we define it as follows:

*We consider a treatment as **essentially placebo** when, in your estimation, any positive treatment effect on the patient’s symptoms is **not** caused by the pharmacological or biological components of the treatment.*

Note that the patient **might still feel better** after undergoing a treatment that is essentially placebo, perhaps because of some nonspecific mechanism or because of the placebo effect. The treatment also **does not have to be completely inert**; it could have an effective pharmacological or biological component for a different complaint than the one the patient currently has. For example, vitamins would usually be essentially placebo when they are prescribed for someone with no vitamin deficiency, but they would **not** when prescribed for someone with the relevant vitamin deficiency. Other examples could be antibiotics when the patient has a viral infection, a gluten-free diet for a patient without a gluten allergy, or traditional placebos such as a sugar pill.

8. Roughly how often do you give treatments that are essentially placebos, on average?

Approximately ______ times per □ 10 years (check what is appropriate)
□ year
□ month
□ week

If you have never prescribed a treatment that was essentially a placebo in your career as a physician, please enter 0 for this question (time scale is irrelevant).

➔ This automatically skips questions **10-12**.

9. Roughly how often do you think your GP colleagues give treatments that are essentially placebos, on average?

Approximately ______ times per □ 10 years (check what is appropriate)
□ year
□ month
□ week

10. Which treatments have you yourself given that would fall under the above definition of essentially placebo treatments? Name up to 5 treatments that come to mind first.

1. ____
2. ____
3. ____
4. ____
5. ____

11. Below are reasons that GPs have given in previous studies for prescribing a treatment that was essentially placebo. When you gave such a treatment, did you do so for any of these reasons? Tick all that apply.

□ To calm the patient
□ To give the patient the feeling that I take them seriously
□ To offer a treatment option in the case of an untreatable illness or symptom

© 2025 Wolters F et al. *JAMA Network Open.*

□ To offer a treatment option in case of allergy or sensitivity to regular treatment
□ To offer a treatment option for non-specific complaints
□ To test whether the patient’s complaints were organic or psychogenic
□ To improve the patient’s symptoms through the placebo effect
□ To comply with the patient’s request for medication
□ To prevent the patient from developing drug addiction or dependence
□ To avoid explanations that would take a lot of time
□ To avoid discontinuing another physician’s prescription
□ Other (please specify):____

12. What did you tell the patient when you gave treatments that were essentially placebos? Tick all that apply.

I said that the treatment...

□ is a treatment.
□ is a placebo.
□ will have no intrinsic/physical/pharmacological effect for their condition.
□ is a treatment with no specific effect for their condition.
□ has worked well for others with their symptoms.
□ stimulates the self-healing process.
□ is a highly effective treatment.
□ is not normally prescribed for their condition, but it might help.
□ may help, and will not hurt.
□ I gave no further information.
□ Other (please specify):____

13. How do you regard your medical training on the use of treatments that are essentially placebos in clinical practice? Has it prepared you sufficiently for cases like those mentioned in this survey? Indicate the degree of sufficiency by indicating a point along the line, with 0 representing ‘wholly insufficient’ and 100 representing ‘wholly sufficient’.

Wholly insufficient Wholly sufficient

0 100

|____|

14. In general, how professionally acceptable do you think it is to prescribe treatments that are essentially placebos to a patient? Indicate the degree of acceptability by indicating a point along the line, with 0 representing ‘completely unacceptable’ and 10 representing ‘completely acceptable’.

Completely unacceptable Completely acceptable

0 100

|____|

© 2025 Wolters F et al. *JAMA Network Open*.

15. When you initiate a treatment with a patient, how often do you present it as **better** (more effective or less side-effects) than you believe it is?

Approximately…

□ Never
□ Rarely
□ About half the time
□ Most of the time
□ (Almost) always

16. When you initiate a treatment with a patient, how often do you present it as **worse** (less effective or more side-effects) than you believe it is?

Approximately…

□ Never
□ Rarely
□ About half the time
□ Most of the time
□ (Almost) always

17. On average, how often do you reassure a patient verbally, without giving a prescription or reference?

□ Never
□ Rarely
□ About half the time
□ Most of the time
□ (Almost) always

Background information

18. Your age: ______ years old

19. Your gender: □ female □ male

20. How many years have you been practicing as a GP?

_________ years

21. How many hours per week do you work in your clinical practice on average?

Approximately _______ hours

22. How many patients do you treat on average per week?

Approximately _______ patients per week

© 2025 Wolters F et al. *JAMA Network Open*.

Opinion or remarks on this survey

21. Do you agree with our definition of ‘treatments that are essentially placebo’ as *“a treatment where any positive treatment effect on the patient’s symptoms is not caused by the pharmacological or biological components of the treatment*”?

◻ Yes ◻ No

22. If you do not agree with our definition, why? **(optional)**

23. Do you have any other comments on the survey? **(optional)**

Thank you very much for your input!

© 2025 Wolters F et al. *JAMA Network Open*.
