# Supplementary material for: Prescriptions of Essentially Placebo Treatments Among General Practitioners in 21 Countries
Source: JAMA Netw Open. 2025 Sep 18;8(9):e2532672. doi: 10.1001/jamanetworkopen.2025.32672 (PMC12447254; doi:10.1001/jamanetworkopen.2025.32672)
Supplement: Supplement 2. — Data Sharing Statement [file jamanetwopen-e2532672-s002.pdf]

Data Sharing Statement

Wolters. Prescriptions of Essentially Placebo Treatments Among General Practitioners in 21 Countries. *JAMA Netw Open*. Published September 18, 2025.
doi:10.1001/jamanetworkopen.2025.32672

Data
Data available: Yes
Data types: Deidentified participant data
How to access data: Data is available upon request. Contact the corresponding author at f.wolters@fsw.leidenuniv.nl.
When available: With publication

Supporting Documents
Document types: None

Additional Information
Who can access the data: Only to researchers whose requests are approved by the study steering group.
Types of analyses: For any purpose.
Mechanisms of data availability: After approval by the steering group and a signed data sharing agreement, as per Leiden University guidelines.
